# A modular and controllable T cell therapy platform for acute myeloid leukemia

**DOI:** 10.1038/s41375-020-01109-w

**Published:** 2021-01-07

**Authors:** Mohamed-Reda Benmebarek, Bruno L. Cadilha, Monika Herrmann, Stefanie Lesch, Saskia Schmitt, Stefan Stoiber, Abbass Darwich, Christian Augsberger, Bettina Brauchle, Lisa Rohrbacher, Arman Oner, Matthias Seifert, Melanie Schwerdtfeger, Adrian Gottschlich, Felicitas Rataj, Nadja C. Fenn, Christian Klein, Marion Subklewe, Stefan Endres, Karl-Peter Hopfner, Sebastian Kobold

**Affiliations:** 1 Center of Integrated Protein Science Munich (CIPS-M) and Division of Clinical Pharmacology, Department of Medicine IV, Klinikum der Universität München, LMU, Munich, Germany; 2 Department of Medicine III, Klinikum der Universität München, LMU, Munich, Germany; 3 Mucosal Immunology and Microbiota Lab, Humanitas Clinical and Research Center, Milan, Italy; 4 Laboratory for Translational Cancer Immunology, Gene Center, LMU Munich, Munich, Germany; 5 Roche Innovation Center Zurich, Schlieren, Switzerland; 6 German Center for Translational Cancer Research (DKTK), Partner Site Munich, Munich, Germany; 7 Einheit für Klinische Pharmakologie (EKLiP), Helmholtz Zentrum München, German Research Center for Environmental Health (HMGU), Neuherberg, Germany; 8 Gene Center, LMU, Munich, Germany

**Keywords:** Targeted therapies, Cancer immunotherapy, Acute myeloid leukaemia

## Abstract

Targeted T cell therapy is highly effective in disease settings where tumor antigens are uniformly expressed on malignant cells and where off-tumor on-target-associated toxicity is manageable. Although acute myeloid leukemia (AML) has in principle been shown to be a T cell-sensitive disease by the graft-versus-leukemia activity of allogeneic stem cell transplantation, T cell therapy has so far failed in this setting. This is largely due to the lack of target structures both sufficiently selective and uniformly expressed on AML, causing unacceptable myeloid cell toxicity. To address this, we developed a modular and controllable MHC-unrestricted adoptive T cell therapy platform tailored to AML. This platform combines synthetic agonistic receptor (SAR) -transduced T cells with AML-targeting tandem single chain variable fragment (scFv) constructs. Construct exchange allows SAR T cells to be redirected toward alternative targets, a process enabled by the short half-life and controllability of these antibody fragments. Combining SAR-transduced T cells with the scFv constructs resulted in selective killing of CD33^+^ and CD123^+^ AML cell lines, as well as of patient-derived AML blasts. Durable responses and persistence of SAR-transduced T cells could also be demonstrated in AML xenograft models. Together these results warrant further translation of this novel platform for AML treatment.

## Keypoints

- Modular platform enabling controlled targeting of AML by SAR-transduced T cells in combination with tandem scFv constructs.
- Efficient lysis of primary AML blasts in vitro and strong antitumoral effects and T cell persistence in xenograft models.

## Introduction

With high relapse rates and few targeted therapeutic options, there is a need develop novel solutions for the treatment of acute myeloid leukemia (AML). While standard therapy (induction chemo- and consolidation therapy) does offer a curative first-line therapy to those eligible [1], leukemic stem cells (LSCs) drive disease relapse in the majority of responders [2]. In spite of significant advances, including allogeneic stem cell transplantation and a growing molecular tailoring of treatment toward driver pathways such as FLT3 [3], the prognosis of relapsed or refractory AML remains poor.

Immunotherapy that promotes the killing of tumor cells by cytotoxic T lymphocytes has entered clinical routine for hematological malignancies in recent years [4–6]. In acute lymphocytic leukemia (ALL), bispecific antibodies utilized for the recruitment of cytotoxic T cells to CD19^+^ leukemic cells have been shown to be an effective approach, and are now part of the standard-of-care [7]. Similarly, anti-CD19 chimeric antigen receptor (CAR) T cell therapy has been approved in ALL and diffuse large B cell lymphoma based on unprecedented response rates [8–10]. The cornerstone of these treatments is a broadly expressed target antigen on tumor cells that is harnessed to redirect T cells toward the cancer or leukemic cell [11]. In the case of B cell neoplasia, the target antigens, CD19 or CD20, are restricted to the B cell lineage, and the potentially adverse side effect of B cell depletion has been manageable [11, 12]. In contrast, myeloid lineage antigens are much less suited as target structures, as the absence of myeloid cells or of major myeloid lineages is associated with a high morbidity and mortality rate [13]. Thus, there is a need to render AML targeting by T cells either modular or conditional to prevent excessive and life-threatening toxicities whilst enabling clinical activity.

CD33 is an antigen expressed in more than 99% of AML cases, therefore offering a targeted therapeutic modality with the potential to induce remission [14, 15]. CD33 is expressed by all early myeloid progenitors (CD34^+^ CD33^+^), thus LSCs that acquired one or more of their transforming events following commitment to the myeloid compartment are targetable [16]. Moreover, CD33 has been shown to be expressed on the majority of CD34^+^ CD38^-^ LSCs of AML patient blasts [17, 18]. Along these lines, CD33-targeting should also be able to eradicate chemo-resistant LSCs. However, antigen negative escape variants cause disease relapse in many targeted therapies, emphasizing the need for sequential or even multiple targeting [19].

The pan-T cell-CD33-targeting bispecific T cell engager (BiTE) AMG330 recently showed encouraging results in a phase I trial [14]. In addition, several AML-specific CAR T cells are currently in clinical trials (NCT03971799; NCT04010877; NCT04156256). However, none of these strategies targeted truly AML-specific antigens but rather antigens either only expressed on subsets of AML cells or co-expressed by normal myeloid progenitors [20, 21]. Although potentially effective, all aforementioned T cell strategies are, once deployed, non-reversible and would therefore benefit from a capability to control T cell activity.

An alternative approach is to transduce T cells with a synthetic agonistic receptor (SAR) composed of an inert extracellular domain (EGFRvIII -referred to as E3) acting as a unique antigen receptor fused to intracellular T cell-activating domains that can be specifically activated by an engineered BiAb [22]. Because SARs have no known natural ligands, this reduces the likelihood of unforeseeable toxicity. Triggering of SAR by the BiAb is dependent on it binding its second specificity, i.e., a selected tumor-associated antigen on the tumor cell. This binding allows for BiAb molecules to aggregate, enabling crosslinking of the SAR. This activates the T cells and directs T cell-mediated lysis [22]. This tumor-killing activity is limited by the supply and half-life of the BiAb. Notably, SAR T cells, unlike CAR T cells, can be removed from the circulation if needed, by using FDA-approved monoclonal antibodies, without having to rely on the addition of a suicide gene [22]. With these favorable properties, SAR T cells developed to target AML could overcome the hurdles of toxicities and escape variants.

To enable better control over T cell activity for AML and ALL indications, we reasoned that we could replace the BiAb (IgG) with tandem scFv (taFv) constructs as these would be more controllable and safer due to their shorter half-life [23, 24]. Here, to test this, we developed novel taFv constructs made up of two scFvs linked by a (G_4_S)_4_ linker. These constructs have dual specificities: one to target AML (via binding CD33 or CD123), and the other to target the SAR-expressing T cell (via binding E3 – which is the inert extracellular domain of the T cell-activating SAR).

We could show that T cells expressing the SAR construct can, in a reversible manner, be selectively activated in the presence of AML cells (CD33^+^ or CD123^+^) and the taFv molecule. We demonstrate that, unlike a conventional BiTE (anti-CD33–anti-CD3), which activates pan-T cells, our E3-specific constructs activate only SAR-transduced T cells – giving additional control over effector cell modifications, phenotype and dosage. Importantly, we highlight substantial activity of the platform in primary AML-blast cultures and in different AML-xenograft models, underpinning the translational potential of the approach.

## Methods

### Animal experimentation

4-week-old female NSG mice (NOD.Cg-Prkdcscid Il2rgtm1WjI/SzJ) were purchased from Charles River (Sulzfeld, Germany). MV4-11-LUC-GFP and THP-1-LUC-GFP xenograft models were established by intravenously injecting 2 ×10^6^ or 10^6^ cells, respectively into the tail vein. taFv molecules were delivered intraperitoneally as indicated. 10^7^ T cells were given intravenously as indicated. All animal experiments were approved by the local regulatory agency (Regierung von Oberbayern). Prior to treatment mice were randomized according to tumor burden. Endpoints were registered by an observer blinded to the treatment groups as previously defined [25]. More than 15% weight loss after experiment start or a decrease in general health condition (decreased mobility, general weakness, hunched posture or ungroomed hair) are defined as humane surrogate endpoints for survival and are later referred to as survival of mice. In vivo imaging approach outlined in supplementary methods.

### Binding studies

Apparent dissociation constants (K_D_) were measured by calibrated flow cytometry on a Guava easyCyte 6HT instrument (Merck Millipore, Burlington, MA, USA) with 3.0 to 3.4 μm Rainbow Calibration particles (BioLegend, San Diego, CA, USA) as calibration control [26]. After normalization, data points were fitted to a one-site specific binding model. Expression and purification of molecules is outlined in supplementary methods.

### Cell lines

PL-21, THP-1, MOLM-13, MV4-11, E.G7-OVA, and SEM cell lines were purchased from ATCC (USA). The E.G7-OVA cell line was modified to express full-length human EGFRvIII (Uniprot Entry P00533 AA 1-29, 298–646), resulting in E.G7-EGFRvIII cells. Luciferase-eGFP (LUC-GFP) overexpressing cell lines PL-21-LUC-GFP, THP-1-LUC-GFP and MV4-11-LUC-GFP were generated according to previously described protocols [22]. Antigen quantification of cell lines are summarized in Supplementary Table 1A. 293Vec-Galv and 293Vec-RD114 were a kind gift of Manuel Caruso, Québec, Canada and have been previously described [27]. All human cell lines were short tandem repeat profiled in house to verify their origin. Cells were used for a time period no longer than two months.

### Cytotoxicity assays

T cells were incubated with tumor cell lines and taFvs at indicated effector-to-target ratios and concentrations. Following a 24 h coculture, the BioGlo Assay (Promega, USA) system was used according to the manufacturer’s protocol.

### Confocal microscopy

Blinded confocal imaging and conjugate quantification were carried out following the selection of 10 representative areas of each slide. Cells in or out of conjugate within each area were quantified and a ratio thereof subsequently determined. For each conjugate, the position of the microtubule organizing center (MTOC) was observed, and its polarization to the immune synapse, or lack thereof, was noted. The ratio of polarized to nonpolarized MTOCs was used to determine the ratio of functional synapses out of all conjugates formed.

### Flow cytometry

Flow cytometry was carried out according to previously published protocols [28]. For cell number quantification CountBright® absolute counting beads (Life Technologies) were added. Samples were analyzed with flow cytometers from BD, Canto II and Fortessa (BD Bioscience, Germany) and a Beckman Coulter CytoFLEX for the long-term cultures. Surface antigen density of cell lines and constructs was evaluated with QIFIKIT (Agilent Dako, Santa Clara, CA, USA). Flow cytometry data were analyzed with FlowJo V10.3 software or GuavaSoft, version 3.1.1 (Merck Millipore). Staining approach outlined in supplementary methods.

### Generation of T cell activating fusion constructs and T cell transduction

SAR construct generation was previously described [22]. SAR-transduced T cells will be referred to as SAR T cells. An anti-CD33–CD28–CD3ζ (anti-CD33 CAR) was generated with the same humanized scFv against CD33 used for the taFv construct [29]. Transduction and expansion of primary human T cells was carried out following a previously described protocol [25]. Virus production methods outlined in supplementary methods.

### Interferon-γ release assays

Human T cell stimulation assays were set up at indicated concentrations and effector-to-target ratios. IFN-γ was quantified by ELISA (BD Bioscience).

### Long-term coculture assays

AML blasts were cultivated for 3 days before coculture. Allogeneic healthy donor T cells were incubated with patient-derived AML blasts at indicated effector-to-target ratios and concentrations. Untransduced T cells were used to control for allogeneic effect. Patient blasts were otherwise cultured according to the previously described protocol [30].

### Patient and healthy donor material

After written informed consent in accordance with the Declaration of Helsinki and approval by the Institutional Review Board of the Ludwig-Maximilians-Universität (Munich, Germany), peripheral blood (PB) or bone marrow (BM) samples were collected from healthy donors and AML patients. At initial diagnosis or relapse, samples were analyzed at the Laboratory for Leukemia Diagnostics of the Klinikum der Universität München as previously described [31, 32]. Patient characteristics are summarized in Supplementary Table 2A, B.

### Statistical analysis

Statistical evaluation was performed using GraphPad Prism software V8.3.1 (San Diego, CA, USA). Differences between experimental conditions were analysed as described in figure P values < 0.05 were considered to be significant. Data are shown as mean values SEM of a minimum of three biological replicates or independent experiments, as indicated. For in vitro experimentation with healthy donor or patient samples, no statistical methods were used to predetermine sample size. These were chosen based on prior experience with this experimental design and patient sample availability. For in vivo experimentation sample sizes were used in accordance with prior experience with the models used.

## Results

### Tandem scFv-mediated effects on SAR T cell activation, proliferation and differentiation

**Fig. 1:**
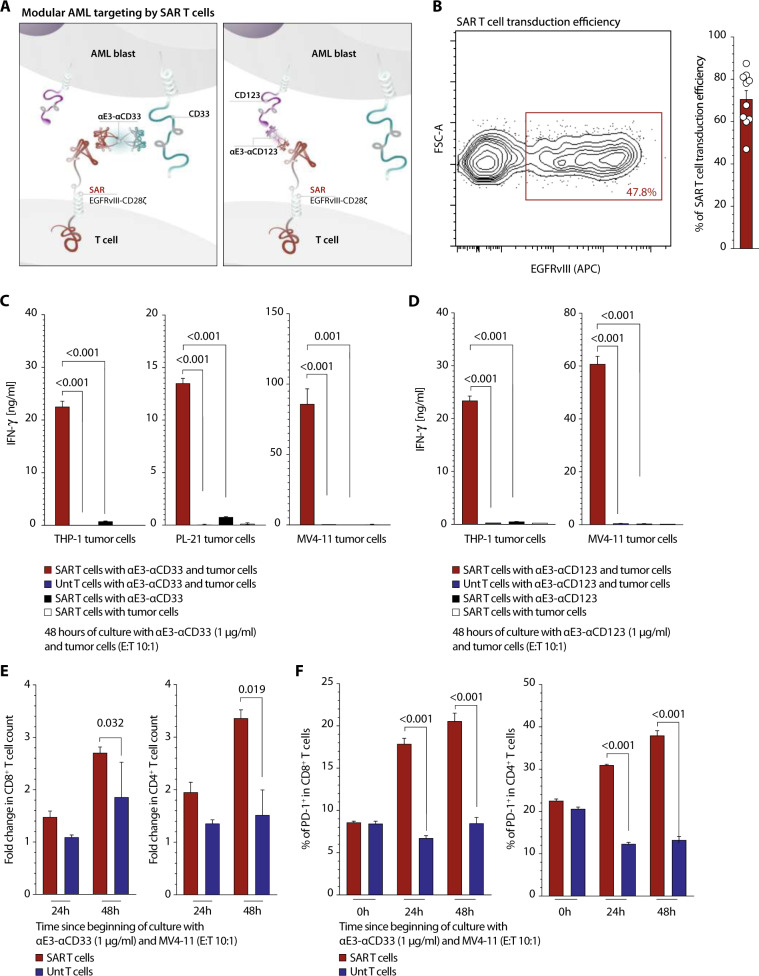
SAR T cells can be bound and triggered by tandem scFvs to induce T cell activation and proliferation. **A** Schematic overview of the SAR construct as well as the modular composition of anti-E3–anti-CD33 and anti-E3–anti-CD123 molecules and CD33 and CD123 target structures. **B** Transduction efficiency flow cytometry plot and SAR expression data in T cells from healthy donors. **C** SAR and unt T cells were cocultured with THP-1, PL-21, or MV4-11 tumor cells and anti-E3–anti-CD33 molecule, with hIFN-γ readout 48 h after coculture. **D** SAR and unt T cells were cocultured with THP-1 or MV4-11 tumor cells and anti-E3–anti-CD123 molecule, with hIFN-γ readout 48 h after coculture. **E** The proliferation rate of the T cells was determined by flow cytometry analysis with surface staining for CD3, CD4, CD8, and EGFR after coculture. **F** SAR and UT T cells were cocultured with MV4-11 tumor cells at a 10:1 E:T ratio. Anti-E3–anti-CD33 taFv was added at a concentration of 1 μg/ml. Readouts were carried out at 0, 24, and 48 h time-points. PD-1 expression of SAR and UT CD4^+^ and CD8^+^ T cells over time (0, 24, and 48 h) is shown. Statistical analysis was performed with unpaired two-tailed Student’s *t* test. Experiments in subfigures (**B**–**F**) show mean values ± SEM and are representative of three independent experiments.

Based on our previous results, we hypothesized that the SAR platform could be developed specifically for AML targeting and treatment [22]. We began by recombinantly generating bispecific anti-E3–anti-CD33 and anti-E3–anti-CD123 taFv molecules. We envisioned that these E3-targeting molecules could efficiently and selectively redirect SAR-expressing T cells to AML blasts (Fig. 1A).

The E3 SAR could be retrovirally transduced into human T cells from healthy donors with high efficiencies (Fig. 1B and Supplementary Table 1B). The novel anti-E3–anti-CD33 molecule was designed to have a high affinity for the target cells (CD33 K_D_ = 19.5 nM), and a lower affinity for the T cells (E3 K_D_ = 235.8 nM) so that aggregates could form more easily on the target cells. The binding properties and apparent dissociation constants of the anti-E3–anti-CD33 molecule to both its targets were analyzed by flow cytometry (Supplementary Fig. 1A and 2A). Similarly, the anti-E3–anti-CD123 molecule was designed using the same backbone as the CD33-targeting one and served as an additional AML-specific targeting taFv molecule to demonstrate the modularity of the platform (CD123 K_D_ = 32 nM) (Supplementary Fig. 1A and 2A). We additionally generated an anti-E3–anti-CD19 molecule to serve as a non-AML-targeting control construct (CD19 K_D_ = 4.9 nM) (Supplementary Fig. 1A and 2A). The anti-CD3–anti-CD33 control has been previously characterized [33]. Purified proteins were analyzed by SDS-PAGE and analytical size exclusion chromatography and protein stability was assessed by fluorescence-based thermal shift assay (Supplementary Fig. 1B to E).

In vitro, taFv-mediated T cell activation is strictly dependent on antibody aggregation on the target cell and their presentation to the T cell in a polyvalent form [34]. To assess this conditional T cell activation upon targeting of the SAR molecule, we incubated SAR T cells with the anti-E3–anti-CD33 construct in the absence or presence of three CD33-expressing AML cell-lines, PL-21, THP-1, and MV4-11, with untransduced (unt) T cells serving as a control. Only SAR T cells in the presence of the taFv construct as well as the target antigen were shown to produce IFN-γ, whereas unt T cells were not stimulated, even in the presence of both taFv and target molecules (Fig. 1C). The anti-E3–anti-CD123 taFv was similarly evaluated, demonstrating both comparable and conditional T cell activation (Fig. 1C, D).

Congruently, SAR T cell activation following coculture with target AML cells resulted in enhanced proliferation of both CD4^+^ and CD8^+^ SAR T cells when compared to other T cell and taFv controls (Fig. 1E). We further observed upregulation of the T cell activation marker PD-1 specifically for SAR T cells compared to the control T cells following coculture with target AML cells and taFv (Fig. 1F). Following activation in culture, SAR T cells were also observed to have a mixture of effector and effector memory phenotypes, similar to the control T cells (Supplementary Fig. 2B)

### SAR T cells form functional immunological synapses to mediate efficient tumor-cell lysis

**Fig. 2:**
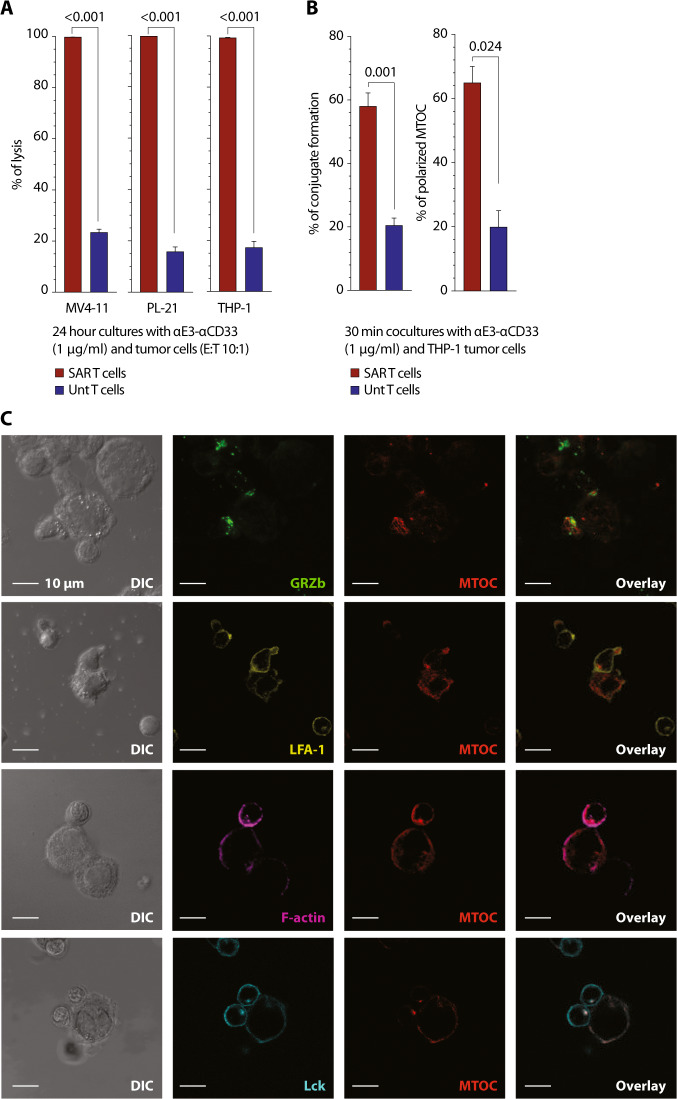
SAR T cells selectively form functional immunological synapses to mediate efficient tumor cell lysis. **A** SAR and unt T cells were cocultured with THP-1, PL-21, or MV4-11 tumor cells with anti-E3–anti-CD33. Following coculture, the BioGlo Luciferase assay was used to calculate the percentage of cells lysed—values shown were normalized to the AML only control condition which was taken as 0 % lysis. **B** SAR or unt T cells were cocultured with THP-1 tumor cells in a V-well plate before transfer to a poly-L-lysine-coated slide. Cells were allowed to adhere for 30 min before fixation and permeabilization. The percentage of T cells conjugated with tumor cells was quantified, as well as the percentage of those conjugates with a polarized MTOC. **C** Double Immunofluorescence labeling was carried out to characterize the polarization of the MTOC, Granzyme B, LFA-1 and F-actin at the SAR T cell IS. For statistical analysis the unpaired two-tailed Student’s *t* test was used. Experiments in subfigures (**A** and **B**) show mean values ± SEM and are representative of at least three independent experiments. Subfigure (**D**) is representative of three independent experiments. Leica TCS SP5 confocal system with a HCX PL APO CS 63x/1.4 oil objective was used for image acquisition on Leica application suite v2.7.3.9723. Tumor cells were GFP positive. Fluorochromes used: MTOC (AF594) Granzyme B (AF647); F-actin (AF647); LFA-1 (AF647); Lck (AF647). For z-axis image reconstruction (stacking) confocal sections were taken 0.2 µm apart.

CD33-expressing tumor cells were effectively targeted and lysed by anti-E3–anti-CD33 and anti-E3–anti-CD123-activated SAR T cells, but not unt T cells (Fig. 2A and Supplementary Fig. 2C). To dissect the mode of action of SAR T cells in these settings, we analyzed the interface between both cell types. Cell conjugates and synapses formed between the T cells and tumor cells were labeled and quantified. SAR T cell conjugates occurred significantly more frequently than unt T cell-target cell conjugates (Fig. 2B). To probe the nature of the immunological synapse (IS), we assessed F-actin and CD11a-LFA-1 accumulation. Strong accumulation of F-actin is indicative of a functional immune synapse, which was observed to span the entire area of the synapse (Fig. 2C). A moderate accumulation of the LFA-1 signal was also seen at the IS, although the signal was also observed across the T cell surface. IS functionality was judged by the polarization of the MTOC, or lack-thereof, as well as the organization pattern of the T cell-associated tyrosine kinase, Lck. Significantly more SAR T cell-target cell conjugates had a polarized MTOC compared to unt T cell control conjugates (Fig. 2B, C). Moderate Lck accumulation was observed at the IS, however a dispersed signal could also be seen (Fig. 2C). SAR T cells also showed granzyme B accumulation and degranulation at the IS, demonstrating formation of a mature and functional IS (Fig. 2C).

### Modular, selective and reversible activation of SAR T cells and their applied safety switches

**Fig. 3:**
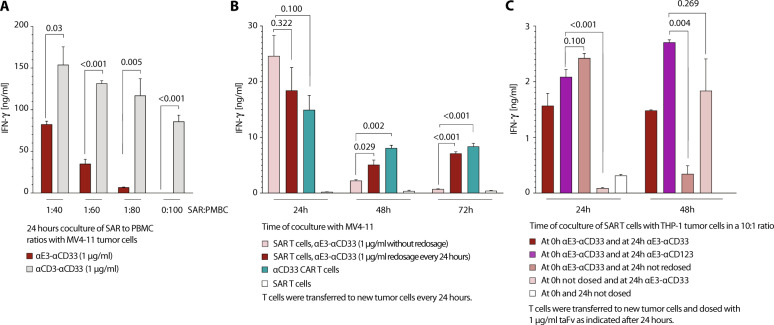
Modular, selective and reversible activation of SAR T cells and their applied safety switches. **A** SAR T cells were serially titrated (1:40, 1:60, 1:80, 0:100) in a PBMC mix. Cells were then cocultured with MV4-11 tumor cells (E:T 10:1), with either a pan-T cell (anti-CD3–anti-CD33, 1 μg/ml) or a SAR-specific molecule (anti-E3–anti-CD33, 1 μg/ml). **B** MV4-11 tumor cells were repeatedly cocultured with SAR T cells with or without redosage of the constructs (1 μg/ml). Anti-CD33 CAR T cells were used as a control and cocultured with tumor cells following the same procedure (no taFv was added) (E:T 10:1). **C** A modularity stress test was carried out using anti-E3–anti-CD33 and anti-E3–anti-CD123 molecules (1 µg/ml). SAR or unt T cells were cocultured with THP-1 tumor cells (E:T 10:1). Readouts were carried out at 24 or 48 h. At assay start, cocultures received either anti-E3–anti-CD33 molecules, anti-E3–anti-CD123 molecules, or no molecules. At 24 h, cocultures were either redosed with the same taFv, redosed with the other taFv against a different target, dosed for the first time with either molecule, or not redosed after initial dosing. At each time point, supernatants were collected and subjected to a hIFN-γ ELISA readout. For statistical analysis, the unpaired two-tailed Student’s *t* test was used. Experiments show mean values ± SEM and are representative of three independent experiments.

Due to the antigen heterogeneity in AML, and because of toxicities associated with the targeting of myeloid lineage antigens, cell therapy approaches need to be modular and controllable [35, 36]. To show selectivity advantages of the SAR platform over BiTE, SAR T cells were serially titrated in a peripheral blood mononuclear cell (PBMC) mix, then cocultured with target cells and either a pan-T cell-targeting molecule (anti-CD3–anti-CD33) or a SAR-specific one (anti-E3–anti-CD33). The selective activation of SAR T cells was evident when the SAR–PBMC mix was cocultured with an anti-E3–anti-CD33 molecule, as IFN-γ levels decreased with lower concentrations of SAR T cells in the mix (Fig. 3A). This titrated T cell activation effect was lost when the anti-CD3–anti-CD33 molecule was employed at equivalent total cell numbers. Furthermore, the anti-E3–anti-CD33 construct did not mediate any T cell activation when incubated with a pure PBMC mix devoid of SAR T cells, whereas the anti-CD3–anti-CD33 molecule was non-selective in activating CD3^+^ T cells in the PBMC mix, as expected (Fig. 3A).

An intrinsic safety switch of the SAR platform is that the activity of SAR T cells is strictly dependent on the presence of the taFv construct. Contrary to CAR T cells, the activity of which is irreversible in the presence of the target antigen, SAR T cell activity should quickly dissipate with clearance of the taFv. Indeed, we found that, following cocultures with MV4-11 tumor cells, SAR T cell activity was reversible over time in the absence of taFv redosing, unlike human anti-CD33 CAR T cells. Importantly, repeated dosing of the taFv molecule could maintain SAR activity at comparable levels to that of the CAR (Fig. 3B). This data indicates that engineering the half-life of the taFv molecule would enable control over SAR activity.

The relatively short half-life of the taFv molecule should also enable modularity of the platform, i.e., the sequential targeting of multiple antigen types that would allow for more refined patient-specific tailoring of the treatment. Modularity was demonstrated when the same SAR T cells were redirected toward AML cells expressing multiple targets. SAR T cells were cocultured with CD33^+^ CD123^+^ THP-1 cells. Through the addition, exchange or depletion of anti-E3–anti-CD33 or anti-E3–anti-CD123-targeting molecules we could show modularity by sequentially redirecting SAR T cells toward different AML targets (Fig. 3C and Supplementary Fig. 3A).

Overall, this approach has the potential to target a multitude of AML-associated antigens with a level of flexibility and controllability that is superior to that of CAR T cells. These advantages together with the aforementioned safety facets make this platform a promising modality for the targeting of myeloid lineage antigens.

### SAR–taFv combination can mediate specific cytotoxicity against patient-derived AML blasts and leukemic stem cells

**Fig. 4:**
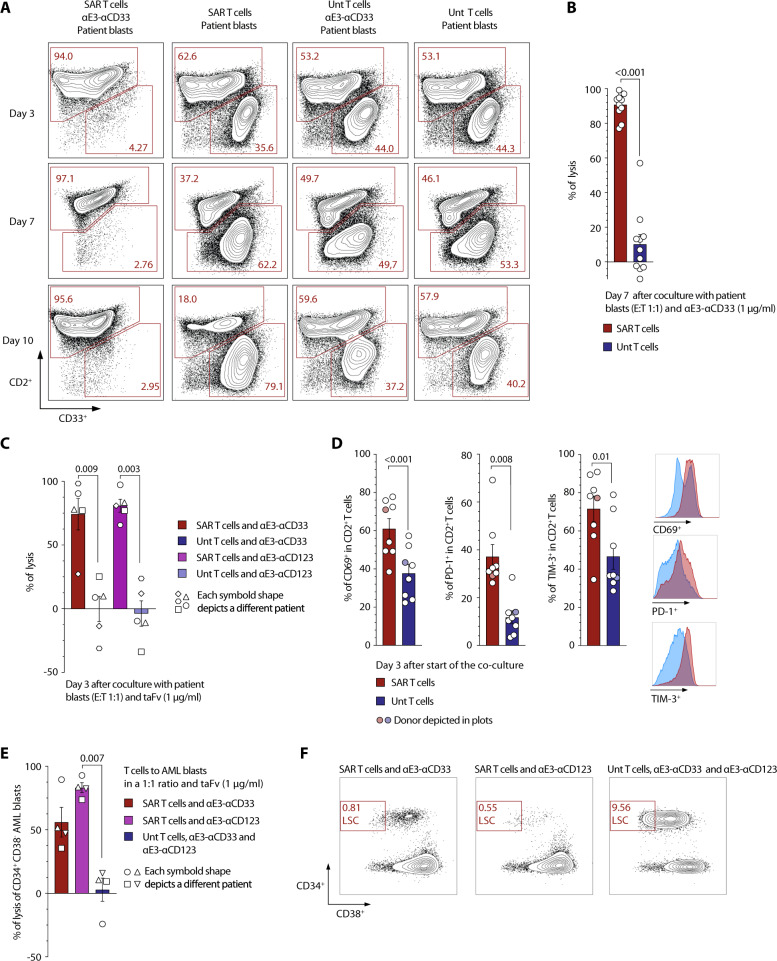
SAR–taFv combination can activate SAR T cells to mediate specific cytotoxicity against patient AML blasts and LSCs. **A** Patient-derived AML blasts targeted by SAR T cells (E:T 1:1) and an anti-E3–anti-CD33 taFv (1 µg/ml), or with controls (SAR T cells and patient blasts, unt T cells with anti-E3–anti-CD33 and patient blasts, unt T cells and patient blasts). In a long-term coculture assay set-up, flow cytometry-based readouts were taken after 3, 7, and 10 days. Cells were stained for CD2 and CD33, to differentiate the T cells and AML blasts respectively. **B** The percentage lysis of patient-derived AML blasts (*n* = 11) by SAR T cells and taFv was calculated as a ratio and compared to unt cells and AML blasts. **C** Patient-derived AML blasts targeted by autologous SAR T cells (E:T 1:1) and either an anti-E3–anti-CD33 taFv (1 µg/ml) or an anti-E3–anti-CD123 taFv (1 µg/ml), or with controls (SAR T cells and patient blasts, unt T cells with either taFv and patient blasts, unt T cells and patient blasts). In a coculture assay set-up, flow cytometry-based readouts were taken after 3 days. Cells were stained for CD2 and CD33, to differentiate the T cells and AML blasts respectively. **D** Following coculture (at day 3), T cells were also stained for CD69, PD-1 and TIM-3. **E** Short-term coculture (18 h) assays were set-up between 5 × 10^5^ patient blasts and SAR T cells (E:T 1:1) and an anti-E3–anti-CD33 (1 µg/ml) or an anti-E3–anti-CD123 (1 µg/ml) molecule, or with controls (SAR T cells only, anti-E3–anti-CD33 and anti-E3–anti-CD123 molecules only, patient blasts only, unt T cells with anti-E3–anti-CD33 and anti-E3–anti-CD123 molecules, unt T cells with AML blasts). To show efficiency of LSC killing, blasts were stained for CD45, CD34, and CD38, and lysis of the CD34^+^CD38^-^ LSC population was quantified as a ratio over unt T cells with patient blasts as a control condition. **F** Representative flow cytometry plots from coculture experiment described in subfigure (**E**). For statistical analysis, the paired two-tailed Student’s *t* test was used. Experiments show mean values ± SEM. Experiments in subfigures (**A**, **B**, and **D**) are representative of six independent long term coculture (LTC) experiments, with multiple patients used per LTC. Experiments in subfigure (**C**) are representative of two independent coculture experiments, with two to three patients used per coculture. Experiments in subfigure E are representative of four independent short term coculture experiments. Patient information for each experiment is listed in supplementary Table 2. CD33 and CD123 patient expression data is depicted in supplementary Fig. 4.

To further translate the potential of our approach, we assessed SAR T cell activity against patient-derived AML blasts. A long-term coculture assay system was utilized to evaluate SAR T cell-mediated cytotoxicity over time. AML blasts were specifically targeted by SAR T cells in the presence of the anti-E3–anti-CD33 molecule, whereas control T cell and taFv combinations were not (Fig. 4A, B and Supplementary Fig. 3B, C). We applied a similar setup to test the efficacy of the approach in an autologous AML patient setting. We could successfully isolate, culture and transduce patient-derived T cells with the SAR (Supplementary Fig. 3D). Their capacity to target their own blasts in the presence of either anti-E3-anti-CD33 or anti-E3-anti-CD123 taFvs was demonstrated, with similar effects to what was already shown in the allogeneic setting (Fig. 4C). SAR T cell activity was also assessed by expression of the markers PD-1, TIM-3, and CD69 after 3 days of coculture. In the presence of the taFv and AML blasts, SAR T cells upregulated PD-1, TIM-3, and CD69 (Fig. 4D and Supplementary Fig. 3E). In addition, we could show that the SAR–taFv combination could also effectively target CD34^+^ CD38^-^ LSC (Fig. 4E, F). The data obtained supports the clinical application of the platform as it shows the efficacy of the approach in targeting patient blasts and LSCs.

### Treatment with the SAR-taFv combination can efficiently eradicate leukemia and enhance survival in vivo

**Fig. 5:**
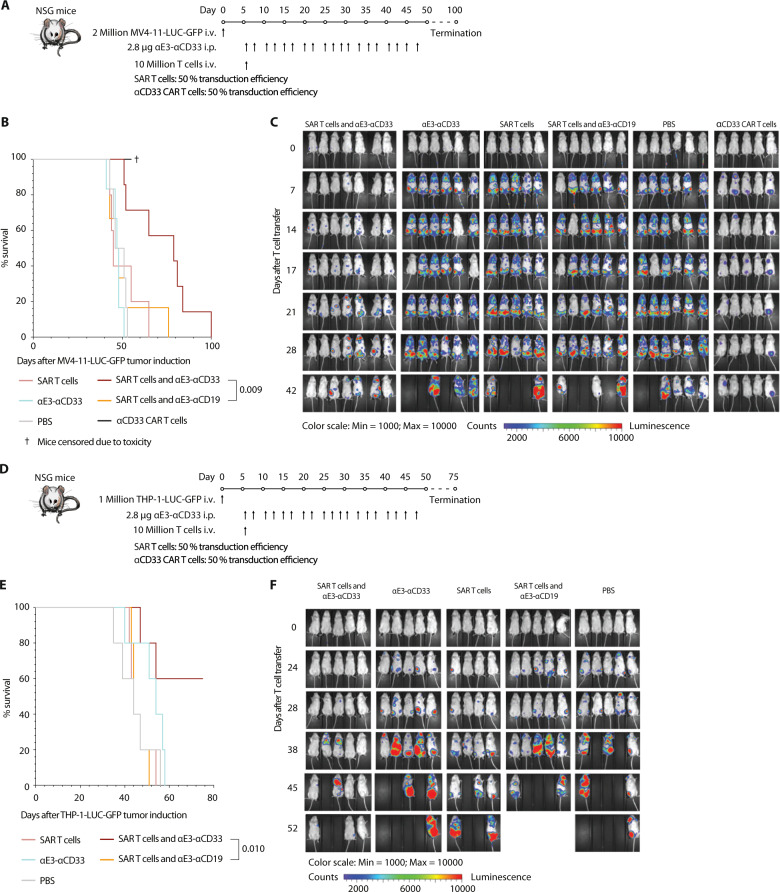
Treatment with the SAR–taFv combination can efficiently eradicate leukemia and enhance survival in vivo. **A** Schematic overview of the experimental setup for (**B** and **C**). NSG mice were inoculated i. v. with 2 × 10^6^ MV4-11-LUC-GFP tumor cells. Mice were treated with a single i. v. injection of T cells. Antibody treatment was given by several i. p. injections of the anti-E3–anti-CD33 molecule (2.8 μg/injection) or a control anti-E3–anti-CD19 molecule (2.8 μg/injection), as indicated by the arrows in the figure. Treatment groups were as follows: SAR T cells and anti-E3–anti-CD33 (*n* = 7), SAR T cells and anti-E3–anti-CD19 (*n* = 6), SAR T cells only (*n* = 5), anti-E3–anti-CD33 only (*n* = 6), PBS (*n* = 6), and anti-CD33 CAR T cells (*n* = 5). **B** Percentage survival readout. † indicates sacrifice of mice suffering from CAR-related toxicity. **C** In vivo imaging data displaying luminescent signal in counts for all experimental groups from treatment day onwards (Days 0, 7, 14, 17, 21, 28, and 42). **D** Schematic overview of the experimental setup for (**E** and **F**). NSG mice were inoculated i. v. with 10^6^ THP-1-LUC-GFP tumor cells. Mice were treated with a single i. v. injection of T cells with or without the anti-E3–anti-CD33 molecule (2.8 μg /injection) or a control anti-E3–anti-CD19 molecule (2.8 μg /injection). Treatment groups were as follows: SAR T cells and anti-E3–anti-CD33 (*n* = 5), SAR T cells and anti-E3–anti-CD19 (*n* = 5), SAR T cells only (*n* = 5), anti-E3–anti-CD33 only (*n* = 5), and PBS (*n* = 5). **F** Percentage survival readout. **G** In vivo imaging data displaying luminescent signal in all experimental groups from treatment day onwards (Days 0, 24, 28, 38, 45, 52). For statistical analysis of survival data, the log-rank test was applied. All in vivo experiments were carried out twice. One representative experiment is shown per xenograft model.

To probe the in vivo function of the SAR–taFv combination, we took advantage of xenograft models of leukemia by engrafting two different AML cell-lines, THP-1-LUC-GFP and MV4-11-LUC-GFP, into NSG mice (Fig. 5A, D). In the MV4-11 model, mice treated with the SAR T cell and anti-E3–anti-CD33 taFv combination experienced major responses to the therapy, with improved tumor control in all treated mice, and a complete response observed in two out of seven mice, which was not seen under any of the negative control conditions. A direct comparison against aCD33-CAR-treated mice was also carried out in this model. While a strong antitumoral response could also be observed in the CAR-treated group, the mice developed severe toxicity (appeared to be non-disease related, likely graft-versus-host disease) and were subsequently taken out of the experiment (Fig. 5B, C). In the THP-1 model, a strong antitumoral response was also observed with the SAR T cell and taFv combination, with one out of five mice clearing the disease (Fig. 5E, F).

Moreover, overall survival was significantly improved in the SAR with anti-E3–anti-CD33 treatment group compared to SAR with anti-E3–anti-CD19 (i.e., non-AML targeting) treatment group in both MV4-11 (*p* = 0.009) and THP-1 (*p* = 0.010) models (Fig. 5B, F). Ex vivo phenotyping of SAR T cells at the experimental endpoint (70 days post transfer) revealed prolonged persistence in the treated mice of the THP-1 model. These SAR^+^ T cells predominantly possessed an effector memory phenotype. CD25 and CD69 staining revealed a higher expression in CD4^+^ and CD8^+^ subsets in the BM compared to the spleen, which correlated with observed tumor burden. PD-1 staining revealed very high expression levels in both the BM and spleen (Supplementary Fig. 4A–D). Together these data indicate that the SAR platform can mediate substantial therapeutic activity in relevant AML xenograft models.

To demonstrate the modularity of the SAR-taFv combination in vivo, we treated THP-1-bearing mice with SAR T cells plus an anti-E3-anti-CD33 or anti-E3-anti-CD123 taFv. We found that mice continuously treated with either taFv showed comparable anti-tumoral efficacy to mice where taFv treatment was switched after four doses, indicative that the targeting moiety can indeed be changed without T cell reinfusion over the course of treatment. In contrast, in mice where taFv treatment was ceased after four doses, the disease quickly progressed, reaching comparable levels to that of mice that received no taFv treatment (Supplementary Fig. 5A, B). This demonstrates the reversibility of T cell activation induced by the taFv modules, which ceases with module decay.

## Discussion

Donor T cell alloreactivity driving the graft-versus-leukemia effect is a major mechanism behind the curative effect of allo-SCT, and supports the notion that T cells are crucial effector cells in the context of AML therapy [37, 38]. Importantly, there is strong preclinical and clinical evidence showing that T cell-based treatment is an effective means of targeting and eliminating AML, including LSCs [39].

Our studies demonstrate that SAR T cells can be redirected by a SAR-specific taFv construct toward the aberrantly expressed AML antigens CD33 and CD123. We could show that SAR T cells are able to specifically recognize multiple targets on several AML cell lines, demonstrating in vitro and in vivo efficacy. We also showed induction of a functional synapse (MTOC polarization, F-actin area), whereas Lck and LFA-1 organization patterns were comparable to those reported for the IS of CAR T cells [40]. This targeted specificity and cytolytic capacity was furthermore demonstrated by the successful targeting of patient-derived AML blasts and LSCs. The potent anti-leukemic activity observed with the SAR platform, in in vivo models and against patient-derived AML blasts, is comparable to those observed in the preclinical testing of AML-targeting BiTEs and CARs [30, 41, 42].

Despite its similarly broad expression on myeloid progenitors and some normal B cell and activated T cell populations, CD33 remains a valuable antigen for the targeting of AML due to its overexpression on blasts in all AML [43, 44]. Low CD33 antigen density in subsets of patient blasts remains a caveat of targeting this antigen [17]. To tackle this, we designed the taFv molecule with a comparably higher CD33 binding affinity, resulting in better targeting of blasts with low CD33 surface expression. A higher affinity for the tumor antigen also means a taFv matrix can be formed on the surface of the AML cells, upon which SAR T cells, with their lower affinity to the taFv, can mediate serial tumor cell killing more efficiently [33, 45]. This design also minimizes antibody trapping in T cell-containing tissues, such as the spleen or lymph nodes, reducing the potential for off-target toxicity [46, 47].

To date, anti-AML CAR T cells have shown limited efficacy in the clinic [48, 49], with on-target off-tumor toxicity being especially problematic in the context of targeting CD33 [50, 51]. To overcome these challenges, highly modular and controllable approaches, as well as those that can make normal hematopoiesis resistant to targeted therapy are needed. One such approach could generate hematopoietic systems unaffected by CD33-targeted therapy [52, 53]. Our SAR platform repurposed for AML, remains, as previously described, highly modular and controllable [22]. Through the direct comparison of a pan-T cell targeting molecule with a SAR-specific one, we could substantiate the claim that nonengineered T cells are not affected by the platform. This level of controllability means the SAR platform distinguishes between two T cell populations in the patient (engineered and nonengineered), which can be carefully selected and tailored. Once the T cell arm of the therapy is adoptively transferred, the redirection and subsequent activation of SAR T cells is completely dependent on the taFv. Clearance of the taFv, in the event of toxicity or on-target-off-tumor activity, would reverse SAR T cell activity. Further or sequential targeting of the AML through the redirection of pre-existing SAR T cells could then be achieved through the introduction of a new taFv with a different AML specificity. In the event of target downregulation as an escape mechanism following treatment (the most prevalent resistance mechanism observed following blinatumomab treatment in ALL patients), platform modularity would again be advantageous. Furthermore, an additional safety layer is ensured by the unique expression of EGFRvIII on SAR T cells (otherwise only expressed on pathologic tissues, such as gliomas), thus depletion with cetuximab as another safety switch is possible if required [22]. Taken together, the SAR platform aligns the advantages of antibody therapy (controllable dosing and reversibility) with that of adoptive T cell therapy (potent anti-tumoral effectors).

Many approaches have emerged attempting to make CAR T cells more modular and controllable. These include but are not restricted to the SUPRA CAR (with tunable availability and affinity) [54], CAR T cells using the synNotch receptor (that can induce transcription of CAR expression to target a second antigen) [55], bispecific CARs (can target two tumor antigens interchangeably) [56] and suicide CAR T cells (such as those using the inducible caspase 9 system, whereby small molecules can activate apoptosis independent of CAR activation) [57]. The SUPRA CAR has the potential to improve the broader applicability and modularity of CAR T cells. The published data however does not show durable in vivo efficacy, while the controllability data (in sparing cells with lower antigen expression) is suboptimal.

Furthermore, its applicability and tailoring toward specific disease settings, such as AML, is yet to be shown [54]. Bispecific CAR T cells are already in clinical testing (anti-CLL-1–anti-CD33; NCT03795779), though given the interpatient LSC diversity, it is unlikely that any two-antigen combination would suffice in eradicating disease across patient cohorts. By comparison, our platform gives more freedom in tailoring a patient-specific combination therapy. As mentioned, the AML setting stands to benefit from improved target selectivity. Application of the synNotch CAR system could improve safety and reduce myeloid toxicity [55]. Despite this, the system still lacks modularity, an important feature for improved targeting of heterogeneous leukemic stem cell populations in AML. A big challenge in the CAR system is autonomous signaling [58]. This is in contrast to the SAR-taFv platform which provides a functionally inert molecule only triggered by the addition of a specific taFv but not by any other known molecule in the body.

Furthermore, many of the modular CAR approaches (such as switchable CAR T cells) rely on the introduction of a neoepitope for selective targeting [59], which comes with immunogenicity issues driving either anti-drug immune responses and dampening activity or potentially triggering toxicities or all of it. SAR T cells and their triggering taFv are fully human or humanizable proteins which markedly reduces immunogenicity risks. Despite the appeal, suicide systems in CARs is a rather brute approach that eliminates all effector cells and requires additional genetic modifications. Importantly, the ability to deplete CAR T cells in the event of toxicities remains to be demonstrated clinically. These approaches have been comprehensively reviewed by Darowski et al. [60].

Perhaps the greatest challenge hindering the success of adoptive T cell therapy in AML is target specificity, which results from disease heterogeneity and diverse antigen expression on LSCs. A recent AML proteomic and transcriptomic study revealed a series of differentially expressed AML-specific antigens, out of which came the rationale that systematic therapeutic combinations would be ideal in the context of AML therapy [18]. This approach was given clinical relevance after a coexpression profile of LSC markers was described for AML patients [17]. The authors found CD33, CD123, CLL1, TIM3, and CD244 to be ubiquitously expressed on AML cells both at diagnosis and relapse stages, and further stressed the benefits of a dual targeting approach for AML. Thus, despite CD33 being expressed on the vast majority of LSCs, the importance of a modular approach with the capacity to simultaneously, or, in the event of antigen escape (or clonal heterogeneity), sequentially target other AML-specific antigens is clear, and is further evidenced by previous work [2, 61].

A strength of our platform—its modularity, stands to benefit from the significant research that has already been carried out on many AML targets as stand-alone targeted therapies [16, 46, 62]. Thus, the repurposing of this knowledge might be a fast and effective method to accelerate the pre-clinical development of the approach. As specific taFv molecules can be tailored individually, the potential for combinatorial approaches will only be limited by the testing and approval of the separate molecules. The SAR platform still stands to benefit from certain optimizations. Amongst these is the modulation of SAR surface expression, an approach that has been successfully applied to the CAR T cell setting [63]. In addition, while advantageous, the short half-life of the taFv will likely require regular infusions, which could present hurdles in the form of practicality and cost.

Collectively, we could comprehensively demonstrate that AML-specific taFvs can be effectively used to target AML in a controllable manner. While further development and more extensive testing are required before its application in a clinical setting, the SAR platform undoubtedly offers new solutions to the ever-challenging AML disease setting.

## Supplementary information

Supplementary Table 2

Supplementary material

Supplementary figure 1

Supplementary figure 2

Supplementary figure 3

Supplementary figure 4

Supplementary figure 5

Supplementary Table 1
