# Supplementary material for: A modular and controllable T cell therapy platform for acute myeloid leukemia
Source: Leukemia. 2021 Jan 7;35(8):2243–57. doi: 10.1038/s41375-020-01109-w (PMC7789085; doi:10.1038/s41375-020-01109-w)
Supplement: Supplementary file 1 — Supplementary Table 2 [file 41375_2020_1109_MOESM1_ESM.pdf]

Supplementary Table 2

A

Figure(s)	Diagnosis	Age (y.o.)	Gender	% blasts	FAB	Cytogenetics	FLT3-ITD mut	NPM1 mut
4A, 4B, 4D	AML	79	M	71	M2	Aberrant complex	-	+
4B, 4D	AML	32	F	96	M5a	Aberrant complex	-	-
4B, 4C, 4D	CML	28	M	58	N.A.	Aberrant complex	-	-
4B, 4D	AML	69	F	92	M1	46,XX	+	+
4B, 4D	AML	78	F	87	M1	46,XX	-	N.A.
4B, 4D	AML	27	F	47	M1	46,XX	-	-
4B, 4D	AML	75	F	N.A.	sAML	46,XX	N.A.	N.A.
4B, 4D	sAML	76	M	76	sAML	N.A.	-	-
4B	AML	75	F	78	M1	46,XX	-	+
4B	AML	51	M	85	M4	46,XY	+	+
4B	AML	71	M	69	M5	47,XY+8/46,XY	-	-
4C, S3D, S3E	AML	56	F	N.A.	M1	Aberrant complex	-	-
4C, S3D, S3E	AML	30	F	N.A.	M5	46,XX,t(8;21)(q22;q22)[10]/46,XX[5]	N.A.	N.A.
4C, S3D, S3E	AML	39	F	N.A.	N.A.	47,XX,+8[15]//46,XX[2]	N.A.	N.A.
4C, S3D, S3E	AML	73	F	N.A.	N.A.	46,XX,inv(7)(q22q31)[4]/47,XX,inv(7)(q22q31),+19[16]	N.A.	N.A.

Disease phase for all patients: Initial diagnosis.

Legend: y.o. years old, % percentage, + positive, - negative, FAB French-American-British classification of acute myeloid leukemia, mut mutation, AML acute myeloid leukemia, sAML secondary acute myeloid leukemia, CML chronic myeloid leukemia, M male, F female, N.A. not available.

B

Patient sample ID	Cytogenetics	FLT3-ITD mut	NPM1 mut
372	Komplex aberrant, incl. 5q13 Del/ETV6-Del/-7/-17	-	-
415	Normal	+	+
491	46,XX,del(7)(q.21)	-	-
573	46,XX,t(5;11)(p.1;q.13)	+	-

Samples from relapsed AML patients used for Figure 4D.
