# Supplementary material for: A modular and controllable T cell therapy platform for acute myeloid leukemia
Source: Leukemia. 2021 Jan 7;35(8):2243–57. doi: 10.1038/s41375-020-01109-w (PMC7789085; doi:10.1038/s41375-020-01109-w)
Supplement: Supplementary file 2 — Supplementary material [file 41375_2020_1109_MOESM2_ESM.docx]

**Supplementary methods**

**Expression and purification**

The anti-E3–anti-CD33 taFv consisted of an M1.1-derived anti-EGFRvIII scFv and a hP67.6-derived anti-CD33 scFv connected by a (G_4_S)_4_ linker^1,2^. An anti-E3–anti-CD19 taFv was generated with a humanized and stabilized ScFv against human CD19^3,4^. An anti-E3–anti-CD123 taFv was generated with a humanized ScFv against human CD123^3,5^. Sequences were cloned into the eukaryotic expression vector pSecTag2/HygroC (Thermo Fisher Scientific) fused to an N-terminal His_6_-tag. Proteins were expressed in Expi293F^TM^ cells (Thermo Fisher Scientific) for 5 to 6 days and purified by nickel affinity and size exclusion chromatography (SEC) using Superdex 200 increase 10/300 columns (GE Healthcare, Little Chalfont, UK) in 20 mM Tris, 300 mM NaCl (pH 9) or 1 x DPBS (Thermo Fisher Scientific) for mouse studies.

**Flow cytometry and microscopy staining**

A fixable viability dye (eFluorTM 780, eBioscience) was included. Antibodies against human CD8a (HIT8a), CCR7 (G04387), CD45RO (UCHL1), PD-1 (EH12.2H7), EGFR (AY13), CD45 (2D1), CD3 (HIT3a), CD4 (OKT4), CD2 (RPA-2.10), CD69 (FN50), TIM3 (F38-2E2).CD34 (561), CD38 (HB-7), and CD123 (6H6) were used (BioLegend). Antibodies against human CD33 (p67.6, Biolegend; WM53, Invitrogen/eBioscience), Granzyme-B (GB11, BD), LFA-1 (CB5.4, BioLegend), anti-alpha tubulin, (DM1A, Abcam) and Lck (28/Lck, BD) were also used.

***In vivo* imaging**

For *in vivo* imaging, mice were injected 10 minutes prior to imaging according to the manufacturer’s instructions (Xenolight D‑Luciferin potassium salt, Perkin Elmer, USA). IVIS Lumina X5 (Perkin Elmer, USA) was used to acquire *in vivo* images. The Living Image Software 4.7.2 was used for analysis (Perkin Elmer, USA).

**Virus production**

For virus production retroviral pMP71 (kindly provided by C. Baum, Hannover) vectors carrying the sequence of the relevant receptor were stably expressed in packaging cell lines 293Vec-Galv and 293Vec-RD114. Using this method, we generated the producer cell lines 293Vec-RD114-E3 and 293Vec‑RD114‑CAR‑CD33.

**Supplementary tables**

**Supplementary table 1: Antigen expression levels of cell lines and SAR expression levels of T cells, as determined by QIFIKIT** (A) Cell line data. (B) SAR T cell data. N.D., not determined. Data is displayed with standard error of the mean as calculated from 3 independent experiments.

**Supplementary Table 2. Patient characteristics** (A) Used for figure 4 and supplementary figure 3. (B) Used for figure 4(D)

**Supplementary figures**

**Supplementary Figure 1. Schematic drawing and overview of constructs and molecules, as well as representative purity and stability testing of molecules** (A) Schematic drawing of taFv molecules, SAR and CAR constructs. (B) Thermostability in 1x PBS as measured by nano-DSF. (C-E) Exemplary purification of anti-E3-anti-CD33 molecule. (C) SDS-PAGE of nickel affinity chromatography. F, flow through; W1 and W2, wash fractions; 1 to 3, elution fractions. (D) Size exclusion chromatography. (E) SDS-PAGE with selected fractions. Ni = pooled fractions after nickel affinity chromatography.

**Supplementary Figure 2. K_D_ determination of scFv modules and SAR T cell activation** (A) Binding to CD33 and CD123 on MOLM-13 cells, CD19 on SEM cells and EGFRvIII on E.G7-EGFRvIII cells, as measured by flow cytometry. K_D_ values are indicated (B) Phenotypic analysis was performed with CD45RO and CCR7 stain and defined as follows: Effector (Eff), CD45RO^-^ CCR7^-^; Effector memory (EM), CD45RO^+^ CCR7^-^; Central memory (CM), CD45RO^+^ CCR7^+^; Naïve, CD45RO^-^ CCR7^+^. (C) SAR and unt T cells were cocultured with THP-1 or MV4-11 tumor cells with anti-E3–anti-CD123 molecule. Following coculture, the BioGlo Luciferase assay was used to calculate the percentage of cells lysed – values shown were normalized to the AML only control condition which was taken as 0 % lysis. For statistical analysis of the unpaired two-tailed Student’s t test was used. Experiments show mean values ± SEM and each are representative of three independent experiments.

**Supplementary Figure 3. Expression data for CD33 and CD123 and cytotoxicity and activation data of patient blasts** (A) Top to bottom: representative flow cytometry plots for MV4-11, THP-1 and PL-21 cell lines. Left to right: CD33 and CD123 expression. (B) Top to bottom: AML patient cells used in short term coculture experiments in Figure 4D. Left to right: CD33 and CD123 expression. (A and B) Blue: isotype control; Red: marker stain. (C) Median fluorescence intensity (MFI) (y axis) of CD33 plotted against % lysis (x axis) for individual patients. (D) Patient-derived T cells were efficiently transduced with SAR construct, with plot depicting SAR expression on transduced patient cells versus untransduced T cells. (E) Following coculture with AML blasts (after three days), autologous T cells (CD2^+^) were stained for CD69, PD-1 and TIM-3, depicted as MFI.

**Supplementary Figure 4. Phenotypic data of adoptively transferred SAR T cells from THP-1 xenograft model** (A) Breakdown of T cell timeline from isolation to experimental endpoint. (B) AML burden in the spleen and bone marrow of surviving mice, represented as tumor cells per bead. (C) Left-to-right: Breakdown of T cell subsets - CD8^+^ and CD4^+^, as well as central memory (CM), effector memory (EM), effector, and naïve. Percentages of aforementioned T cell subsets within the spleen and bone marrow of the treated group (SAR T cells with anti‑E3–anti-CD33 taFv). Inner circle: CD4/CD8 ratios. Outer circle: cell phenotypes within each subset. (D) Activation of EM T cells shown as frequency of parent, in the bone marrow and spleen of treated mice. Left to right: CD25 expression in CD4^+^ and CD8^+^ T cells. CD69 expression in CD4^+^ and CD8^+^ T cells. PD-1 expression in CD4^+^ and CD8^+^ T cells. For statistical analysis the paired two-tailed student’s t test was used. Experiments show the numbers of adoptively transferred T cells and are representative of 3 treated mice from one *in vivo* experiment.**Supplementary Figure 5. SAR-taFv treatment is modular and controllable**

***in vivo*** (A) Schematic overview of the experimental setup for (B and C). NSG mice were inoculated i. v. with 10^6^ THP-1-LUC-GFP tumor cells. Mice were treated with a single i. v. injection of T cells with or without the anti-E3–anti-CD33 molecule (2.8 μg/injection) or an anti-E3-anti-CD123 molecule (2.8 μg /injection). Antibody dosage was varied between groups to demonstrate the reversibility of SAR T cell activation upon cessation of taFv treatment, as well as redirection of SAR T cells towards alternative targets via switching of the tumor targeting taFv molecule. Treatment groups were as follows: SAR T cells only (n = 5), SAR T cells and anti-E3–anti-CD33 (4 doses only – cessation) (n = 5), SAR T cells and anti-E3–anti-CD123 only (4 doses only – cessation) (n = 5), SAR T cells and anti-E3–anti-CD33 (12 doses of anti-E3–anti-CD33 – continuous), SAR T cells and anti-E3–anti-CD123 (12 doses of anti-E3–anti-CD123 – continuous), SAR T cells and anti-E3–anti-CD33 (4 doses of anti-E3–anti-CD33 followed by 8 doses of anti-E3–anti-CD123 – switch) (n = 5), SAR T cells and anti-E3–anti-CD123 (4 doses of anti-E3–anti-CD123 followed by 8

doses of anti-E3–anti-CD33 – switch), (B) *In vivo* imaging data displaying luminescent signal in all experimental groups from treatment day onwards (Days 0, 4, 7, 11, 14, 18 and 25).

1. Herrmann M, Krupka C, Deiser K, et al. Bifunctional PD-1 × αCD3 × αCD33 fusion protein reverses adaptive immune escape in acute myeloid leukemia. *Blood*. 2018; 132(23):2484-2494.

2. Kuan C-T, Wikstrand CJ, Archer G, et al. Increased binding affinity enhances targeting of glioma xenografts by EGFRvIII-specific scFv. *International Journal of Cancer*. 2000; 88(6):962-969.

3. Kügler, M, Stein C, Schwenkert M, et al. Stabilization and humanization of a single-chain Fv antibody fragment specific for human lymphocyte antigen CD19 by designed point mutations and CDR-grafting onto a human framework. Protein Engineering, Design and Selection, 2009. 22(3):135-147.

4. Schubert I, Kellner C, Stein C, et al. A single-chain triplebody with specificity for CD19 and CD33 mediates effective lysis of mixed lineage leukemia cells by dual targeting. *mAbs*. 2011; 3(1):21-30.

5. Stein C, Kellner C, Kügler M, et al. Novel conjugates of single-chain Fv antibody fragments specific for stem cell antigen CD123 mediate potent death of acute myeloid leukaemia cells. *British Journal of Haematology*. 2010; 148(6):879-889.
