# Supplementary figures and images for: A modular and controllable T cell therapy platform for acute myeloid leukemia
Source: Leukemia. 2021 Jan 7;35(8):2243–57. doi: 10.1038/s41375-020-01109-w (PMC7789085; doi:10.1038/s41375-020-01109-w)

Supplementary Figure 1

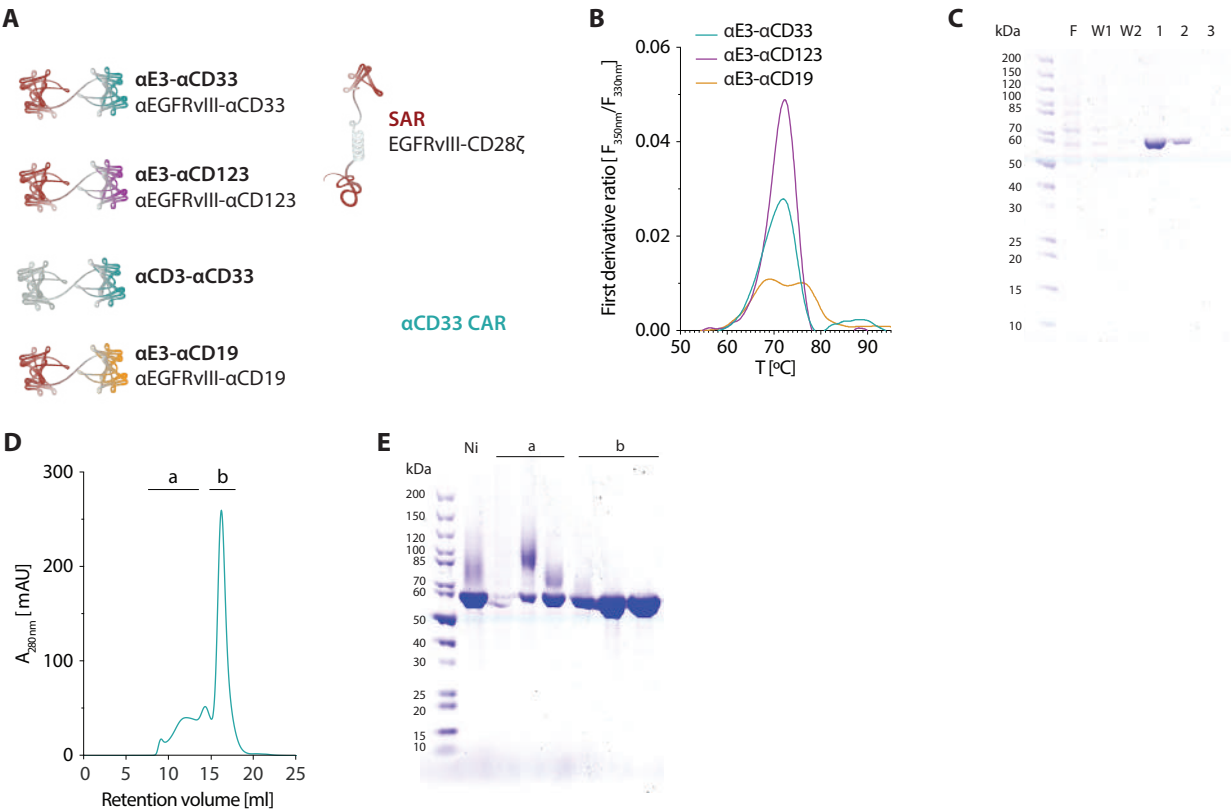

Supplement: Supplementary file 3 — Supplementary figure 1 [file 41375_2020_1109_MOESM3_ESM.pdf]

Supplementary Figure 2

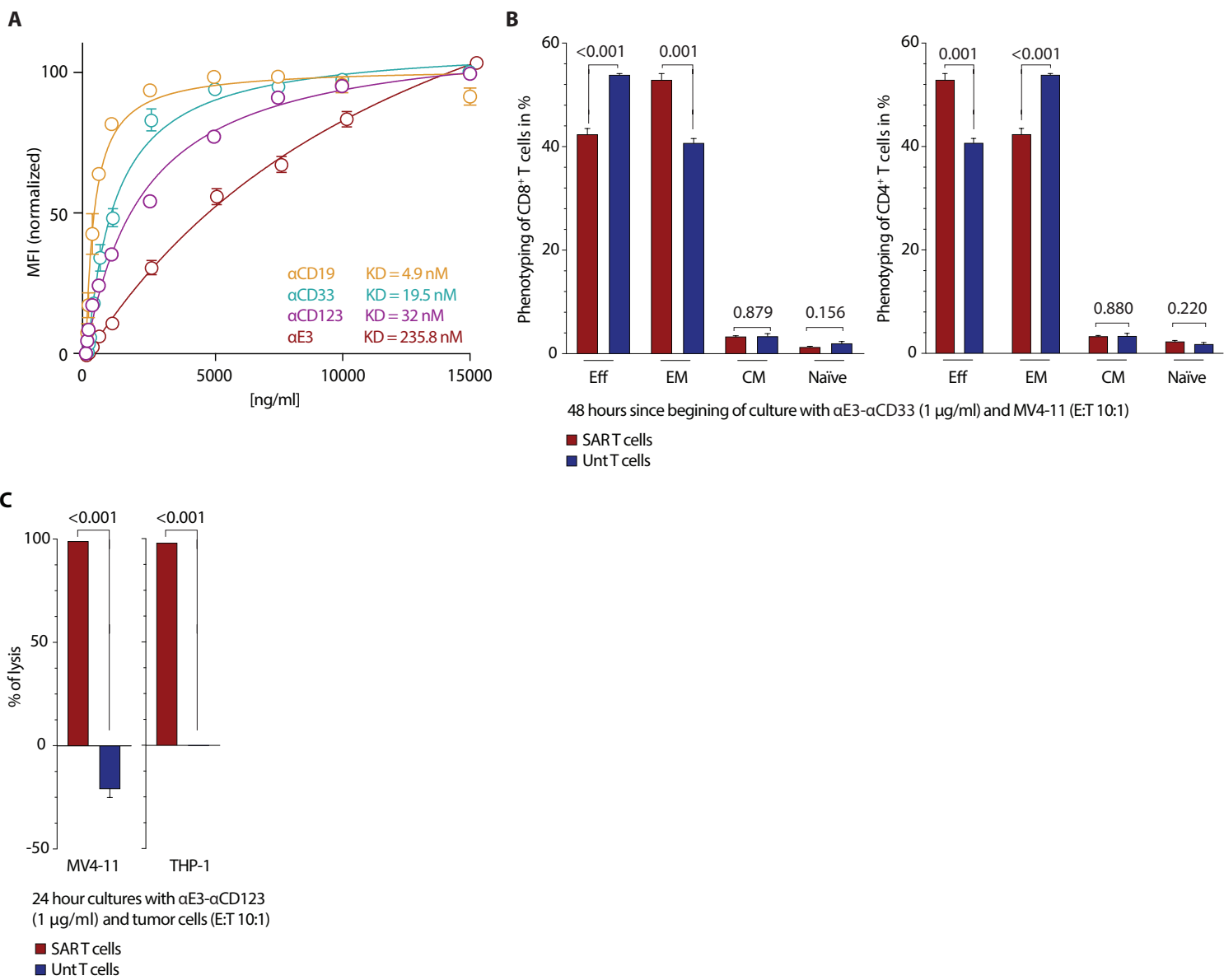

Supplement: Supplementary file 4 — Supplementary figure 2 [file 41375_2020_1109_MOESM4_ESM.pdf]

Supplementary Figure 3

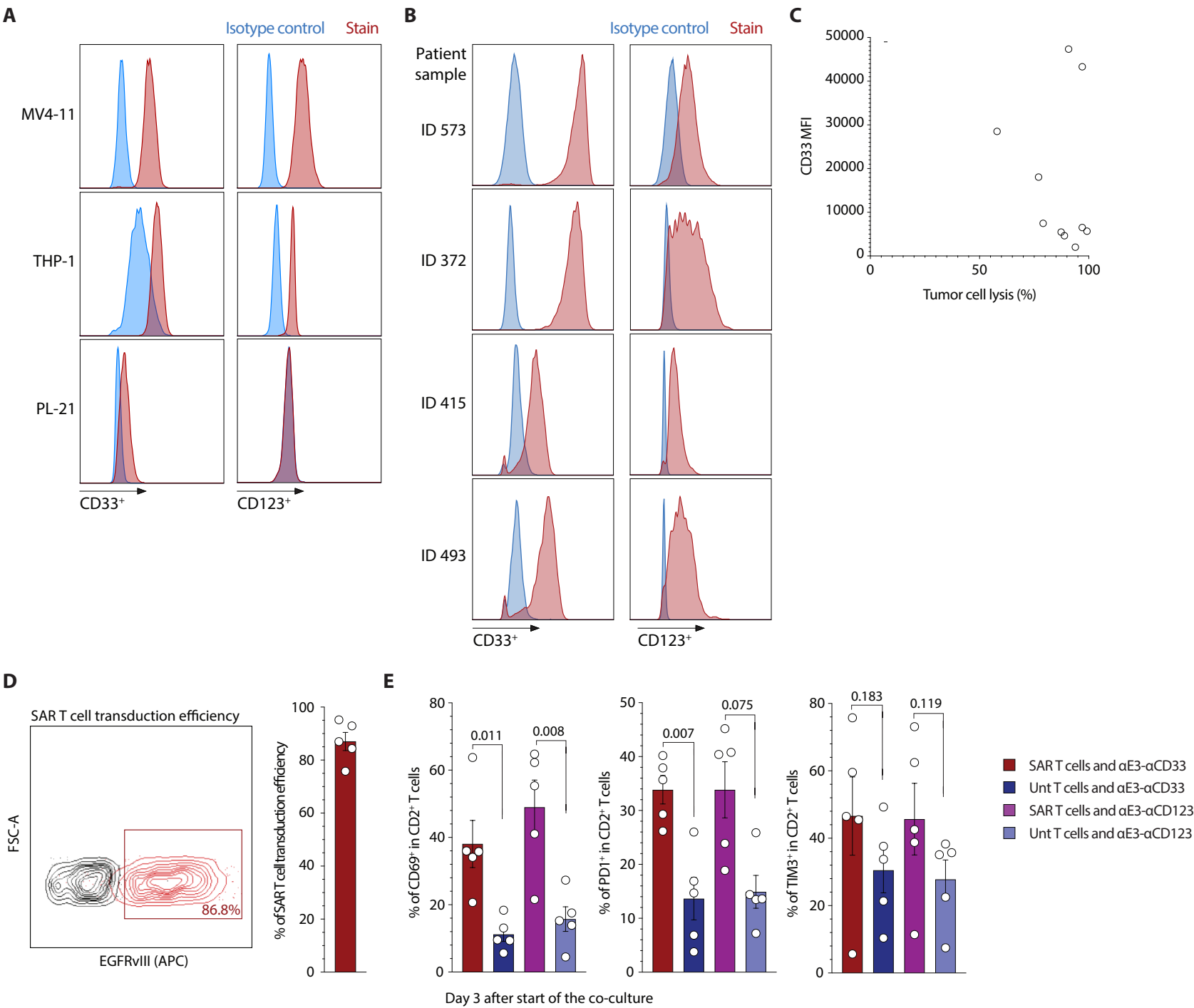

Supplement: Supplementary file 5 — Supplementary figure 3 [file 41375_2020_1109_MOESM5_ESM.pdf]

Supplementary Figure 4

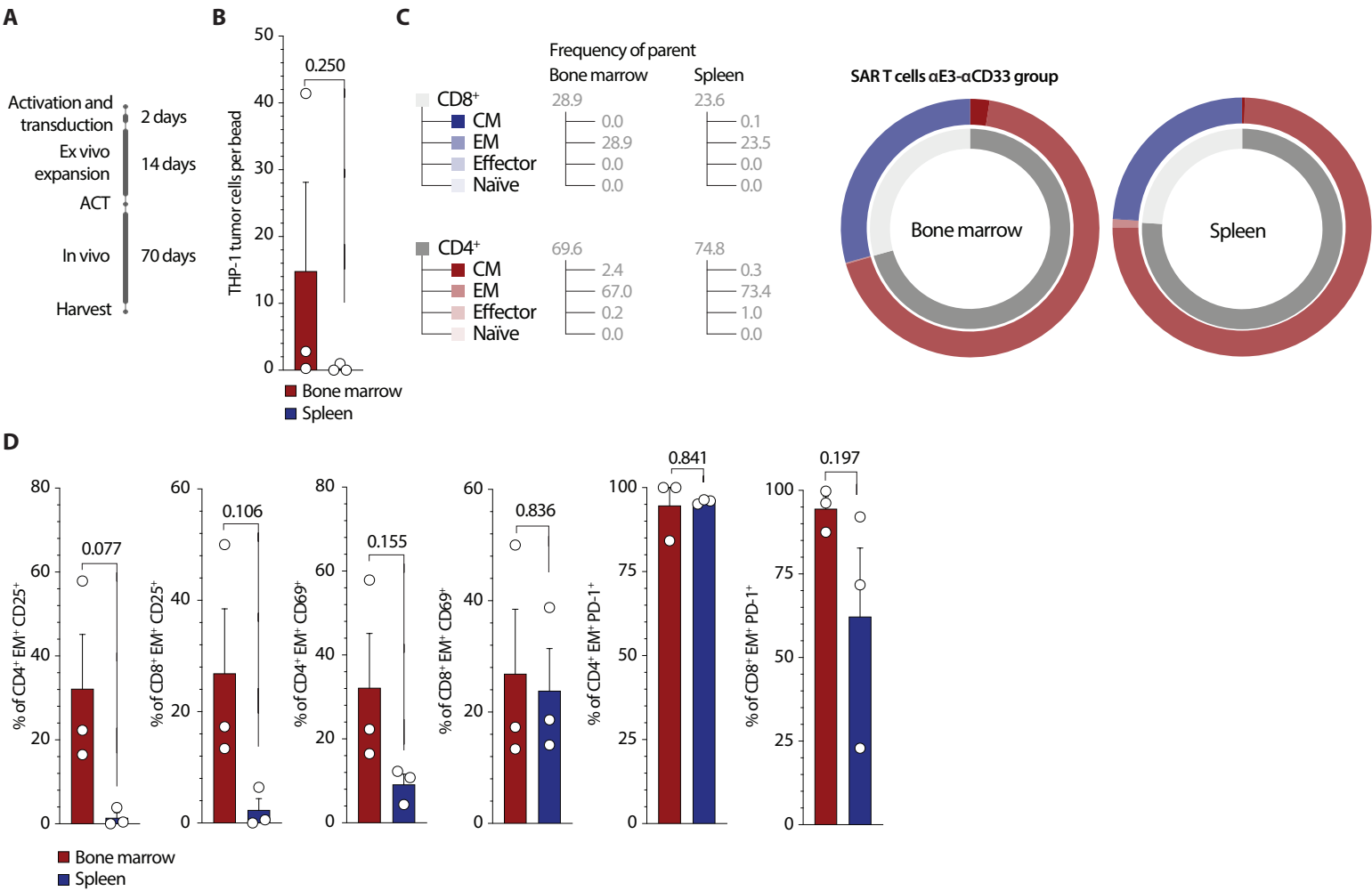

Supplement: Supplementary file 6 — Supplementary figure 4 [file 41375_2020_1109_MOESM6_ESM.pdf]

Supplementary Figure 5

A

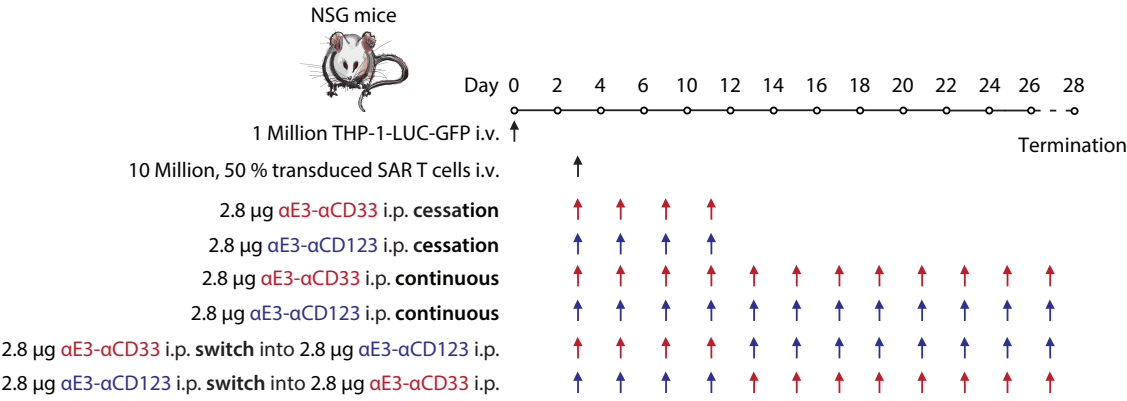

B

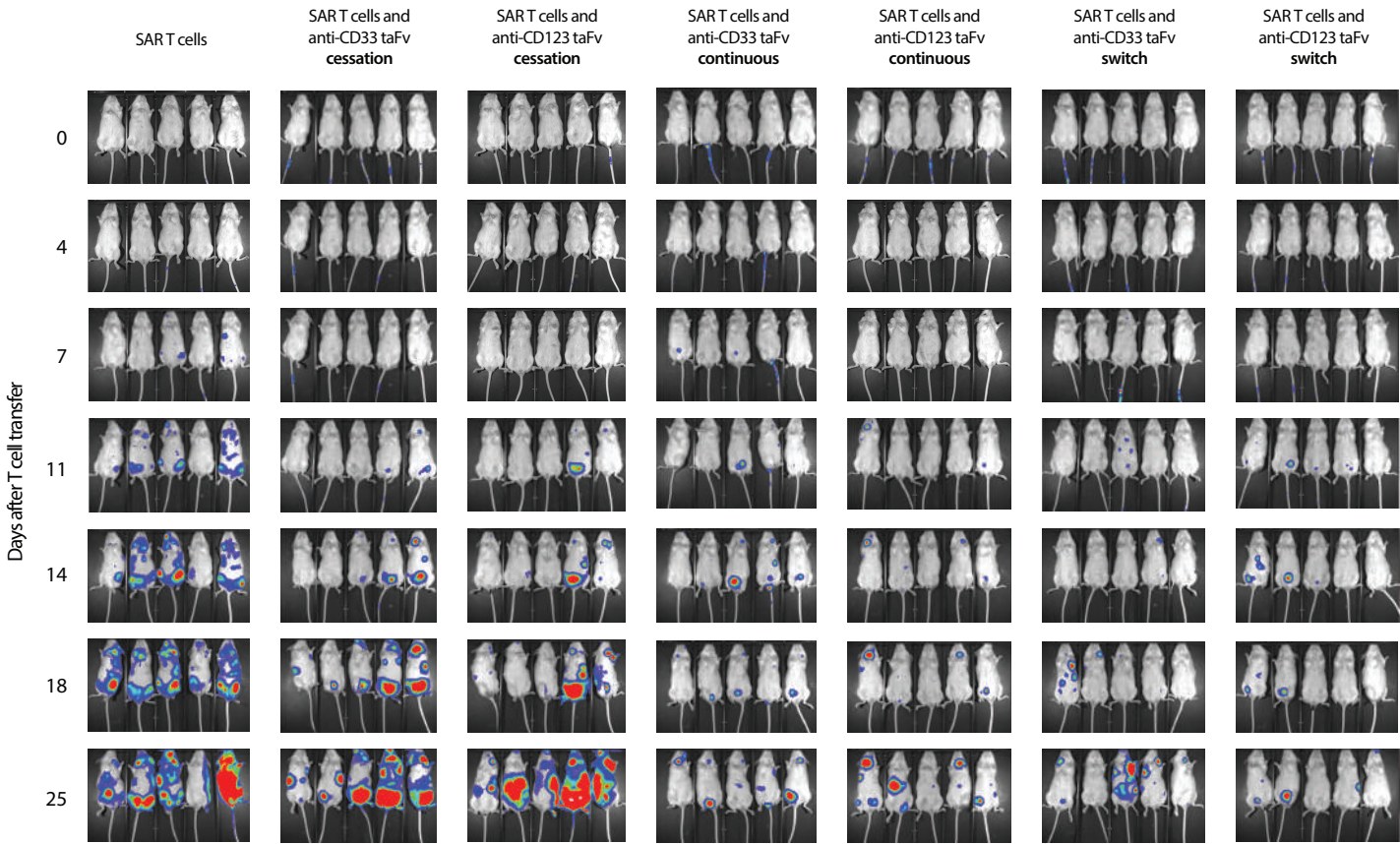

Supplement: Supplementary file 7 — Supplementary figure 5 [file 41375_2020_1109_MOESM7_ESM.pdf]
