# Supplementary material for: A modular and controllable T cell therapy platform for acute myeloid leukemia
Source: Leukemia. 2021 Jan 7;35(8):2243–57. doi: 10.1038/s41375-020-01109-w (PMC7789085; doi:10.1038/s41375-020-01109-w)
Supplement: Supplementary file 8 — Supplementary Table 1 [file 41375_2020_1109_MOESM8_ESM.pdf]

Supplementary Table 1

A

**Cell lines**	**CD33** [Absolute molecule count ± SEM]	**CD123** [Absolute molecule count ± SEM]
**MOLM-13**	69000 ± 8000 (Ref. 33)	12468 ± 134
**THP-1**	40858 ± 954	12671 ±167
**MV4-11**	51695 ± 707	10819 ±2258
**PL-21**	N.D.	N.D.

B

**Transduced T cells**	**EGFRvIII** [Absolute molecule count ± SEM]
**SAR**	105061 ± 1045
